# Benefit of Modulated Masking in hearing according to age

**DOI:** 10.1016/j.bjorl.2024.101487

**Published:** 2024-08-08

**Authors:** Mônyka Ferreira Borges Rocha, Karina Paes Advíncula, Cristiane do Espírito Santo Xavier Simões, Diana Babini Lapa de Albuquerque Britto, Pedro de Lemos Menezes

**Affiliations:** a Universidade Federal de Alagoas, Programa de Pós-Graduação em Biotecnologia, Maceió, AL, Brazil; b Universidade Federal de Pernambuco, Departamento de Fonoaudiologia, Recife, PE, Brazil; c Universidade Federal de Pernambuco, Curso de Fonoaudiologia, Recife, PE, Brazil; d Universidade Federal Rural de Pernambuco, Programa de Pós-Graduacão em Biotecnologia, Recife, PE, Brazil; e Universidade Estadual de Ciências da Saúde de Alagoas, Departamento de Fonoaudiologia, Maceió, AL, Brazil

**Keywords:** Electrophysiology, Evoked potentials auditory, Speech perception, Perceptual masking, Aging

## Abstract

**Objective:**

To analyze the Benefit of Modulated Masking (BMM) on hearing in young, adult and elderly normal-hearing individuals.

**Methods:**

The sample included 60 normal-hearing individuals aged 18–75 years who underwent behavioral assessment (sentence recognition test in the presence of steady and modulated noise) and electrophysiological assessment (cortical Auditory Evoked Potential) to investigate BMM. The results were analyzed comparatively using the paired *t*-test and ANOVA for repeated measures, followed by the Bonferroni post-hoc test (*p*-value < 0.05).

**Results:**

A decrease in latencies and an increase in amplitudes of cortical components (P1-N1-P2) was observed due to noise modulation in all age groups. Modulated noise generated better auditory threshold responses (electrophysiological and behavioral), compared to steady noise. The elderly presented a higher threshold in both hearing domains, compared to the other participants, as well as a lower BMM magnitude.

**Conclusion:**

It was possible to conclude that the modulated noise generated less interference in the magnitude of the neural response (smaller latencies) and in the neural processing time (larger amplitudes) for the speech stimulus in all participants. The higher auditory thresholds (electrophysiological and behavioral) and the lower BMM magnitude observed in the elderly group, even in the face of noise modulation, suggest a lower temporal auditory performance in this population, and may indicate a deficit in the temporal resolution capacity, associated with the process of aging.

**Level of evidence:**

3.

## Highlights

• Less disturbance from modulated noise in the magnitude and time of neural processing.
• Modulated noise generated lower electrophysiological and behavioral thresholds.
• Elderly people had higher electrophysiological and behavioral thresholds.
• Lower BMM magnitude in the elderly suggests lower temporal auditory performance.

## Introduction

Temporal Auditory Processing (TAP) consists of one of the fundamental mechanisms of hearing with regard to the process of understanding speech in silent, noisy and/or reverberant environments, since this processing plays an important role in the perception of the temporal characteristics of a sound or its changes within a period of time.^1^, ^2^

Studies point to an important relationship between TAP and senescence, since elderly individuals have complained of difficulties in understanding speech in the absence of changes in auditory threshold patterns. Such discussions have influenced the increase in research into the investigation of temporal and aging processes.^3^

One of the phenomena related to the temporal processing ability of hearing concerns the Benefit of Modulated Masking (BMM), also known as Masking Release.

This phenomenon is related to the listener's temporal auditory ability to identify audible signals of a sound in the face of temporal fluctuations of the masking noise,^4^, ^5^ enabling the detection and decoding of speech signals, and consequently, better performance in recognizing the target sound.^6^, ^7^

Studies using psychoacoustic tests with the aim of investigating BMM in the behavioral domain, showed a typically lower speech perception threshold when faced with modulated masking compared to constant/stable masking,^8^, ^9^, ^10^ and when researched in different age groups, a lower magnitude of this phenomenon in elderly individuals, even in the presence of normal peripheral hearing, with this reduction in BMM being attributed to a deficit in temporal auditory processing in these individuals.^11^, ^12^

The use of electrophysiological tests, such as the Cortical Auditory Evoked Potential (CAEP) has been considered an objective measure for evaluating BMM, providing complementary data to behavioral tests evaluating temporal processing and enabling a neuroelectric evaluation of the auditory cortex in the face of this temporal phenomenon.^13^, ^14^

From this perspective, the study of the BMM phenomenon in the electrophysiological and behavioral domains has enabled the improvement of research regarding TAP abilities, and the establishment of parameters for analyzing speech recognition in noise situations, since electrophysiological tests enable determine whether such objective measures are predictive of auditory behavioral performance.^5^

Considering the importance of investigating BMM and obtaining parameters for analyzing this phenomenon related to temporal processing abilities, it is essential to carry out studies on behavioral and electrophysiological tests of hearing in different age populations, making it possible to observe the temporal auditory processing capacity related to speech perception in noise as a function of age. In view of this, this study aims to analyze the BMM on hearing in young, adult and elderly normal-hearing individuals.

## Methods

This analytical, cross-sectional-observational study was developed at the Audiology Laboratory of the Speech Therapy Department of the Federal University of Pernambuco (UFPE) from August 2022 to June 2023, with approved by the Ethics and Research Committee in Beings (number 5.140.668).

Individuals between 18 and 75 years without complaints of hearing loss were included, and based on detailed anamnesis, individuals with a history of neurological and/or psychiatric diseases, cognitive deficits and malformations of the ear pinna and external auditory canal were excluded. A cognitive screening test (Montreal Cognitive Assessment Test – MoCA)^15^ was also performed.

The presence of changes and/or malformations in the external and/or middle ear was ruled out by inspecting the external auditory canal and performing an immitaniciometry exam (226 Hz probe), with the presence of tympanometric curve A and the presence of ipsi and contralateral reflexes.^16^, ^17^ In the audiometry examination, normal hearing was considered to be the presence of a quadritonal average of less than 20 dB HL in both ears, considering the frequencies of 500 Hz, 1000 Hz, 2000 Hz and 4000 Hz.^18^ The normality score in the MoCA test was ≥ 26 points.^15^

Although there was no matching of age groups based on the participants' education, this condition was minimized due to the absence of disparity in cognitive abilities between young people, adults and elderly people, observed in the evaluation with the MoCA test.

### Sentence recognition in noise test

To investigate BMM in the behavioral domain, the Sentence Recognition in Noise Test was carried out, using 12 lists (with 20 sentences each) from the Hearing In Noise Test (HINT) – Brazil,^19^ to obtain the Sentence Recognition Threshold (LRS) in modulated and stable noise conditions.

Participants, sitting in an armchair inside a soundproof booth, were instructed to repeat the test sentences as they heard, ignoring the presence of competing noise. The sentences and competitive background noise were sent to the right ear via a supra-aural earphone (Sennheiser HD580). Response monitoring was carried out using Matlab software (Matrix Laboratory®), version R2012a.

The HINT sentences consisted of everyday phrases, presented to the participant once in order to exclude learning bias, with intensity initially higher than the expected recognition threshold, being 60 dB SPL in the modulated noise condition and 70 dB SPL in the noise condition steady.^19^ The exact repetition of the phrase was established as a criterion for success.

The competitive Speech-Shaped Noise (SSN) was presented randomly in the steady condition with an intensity of 65 dB SPL and in the modulated condition, with intensity variation between 65 dB and 30 dB SPL and modulation rate of 10 Hz.^4^ Noise modulation was produced using the Tucker-Davis Technologies-RX6 (TDT-RX6) acoustic signal processor.^10^

#### SRT research

To obtain the LRS, the transformed descending-ascending method (two down ‒ one up)^20^ was used, where for every two consecutive correct answers, the signal intensity decreased by 2 dB in the following sentence, and for each incorrect answer, the intensity of Presentation of the following sentence was increased by 2 dB.

Each individual's final SRT was obtained by averaging three threshold survey measurements in dB SPL (for both types of noise), in order to consider the reproducibility of the behavioral threshold. The BMM was defined through the difference between the SRT in the presence of steady noise (adopted as a reference) and the SRT in the presence of modulated noise.

### Cortical auditory evoked potential

The investigation of the BMM in the electrophysiological domain was carried out by obtaining the CAEP (Intelligent Hearing Systems – IHS), evoked simultaneously by a Synthetic Speech Stimulus /ba/ and competitive Speech Noise (SSN) in a steady and modulated condition.^21^ Participants remained seated in an armchair inside a soundproof booth, watching a video without audio and instructed not to sleep. Four electrodes were positioned in the following configurations: two reference electrodes (negative polarity) on the right (A1) and left (A2) earlobes; an electrode (positive polarity) on the vertex of the head (Cz) and a ground electrode on the lower region of the Forehead (Fpz). The speech stimulus /ba/ and the competitive noise were presented via Earphones (ER2) monaurally to the participants' right ear.

To acquire CAEP, the /ba/ stimulus was presented in a modified waveform (rate of 24,414 Hz), compatible with the digital signal of the TDT-RX6 platform and calibrated with reference to the dB SPL of a 1 kHz continuous tone, equivalent peak (dB NPSpe). The /ba/ stimulus lasted 80 milliseconds (ms) and was presented at a fixed intensity of 65 dB SPLpe and a rate of 3.8 stimuli per second.

The competitive noise was presented next to /ba/ in three different and random conditions: stable noise with an intensity of 30 dB NPSpe (weak steady noise); stable noise with an intensity of 65 dB NPSpe (strong steady noise); modulated noise at intensities ranging between 30 and 65 dB NPSpe at a frequency of 25 Hz.

Acquisition of potentials and research of the electrophysiological threshold

The cortical recordings were analyzed individually, blindly, by three evaluators, to identify and mark the potentials (P1, N1 and P2) in the three competitive noise conditions, considering the measures of latency (ms), amplitude (microvolts-µV) and morphology. The P1 potential was identified as the most robust first positive wave at around 50 ms; the valley with the greatest negativity subsequent to the P1 component was identified as the N1 potential; and the P2 response was considered as the most robust positive wave after N1.

The investigation of the CAEP electrophysiological threshold with /ba/ stimulus was carried out in strong modulated and stable noise conditions, starting from the reduction of the intensity of the /ba/ stimulus from 10 to 10 dB until the disappearance of the P1-N1-P2 complex; then the intensity was increased by 5 dB until its appearance, this intensity being the result of the threshold. The magnitude of the BMM was measured based on the difference between the electrophysiological thresholds in the modulated and stable noise conditions, in decibels (dB NPSpe).

### Statistical analysis of data

The results were analyzed statistically (Statistical Package for the Social Sciences – SPSS; version 20.0). The samples per group followed the normal distribution, according to the Shapiro-Wilk test and use of Hair et al.'s^22^ criteria for analysis of asymmetry (−2 and +2) and kurtosis (−7 and +7). The analysis of the results of the cortical components in each noise group was performed using the ANOVA test for repeated measures, followed by the Bonferroni post-hoc test (*p*-value < 0.05). The paired *t*-test was used for comparative analysis of the electrophysiological and behavioral thresholds between the steady and modulated noise groups (*p*-value < 0.05).

## Results

Initially, 70 individuals participated in the study, with 10 excluded from the final sample, due to occlusion of the external auditory canal (3), thresholds compatible with mild/moderate hearing loss (5) and for not attending the behavioral and electrophysiological examination (2). The final sample was made up of 60 normal-hearing individuals organized into three age groups: “Young-adult” group (15 women and 5 men) with a minimum age of 18, a maximum of 30 and an average of 22.8 years; “Adult” group (10 women and 10 men) with a minimum age of 31, a maximum of 50 and an average age of 37.7 years; “Elderly” group (14 women and 6 men) with a minimum age of 60, a maximum of 75 and an average age of 64.7 years.

**Figure 1:**
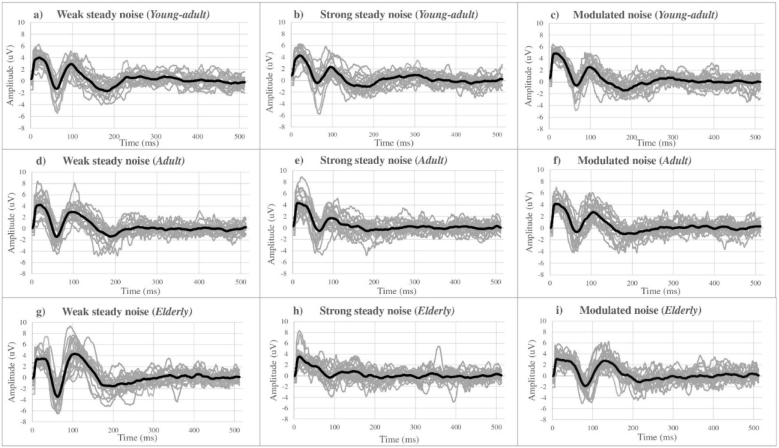
Grand averages of CAEP waves for the three masking conditions in each age group. Legend: CAEP cortical waves for each participant (light gray) and grand average by age group (black).

Fig. 1 presents the individual responses of the CAEP waves and the general average (grand average) of the waves in each age group and for each type of masking noise used. Each participant's cortical waves are shown as light gray lines and each group's average waves are shown as heavy dark lines. It is possible to observe that the cortical waves in the strong stable noise condition have a smaller magnitude, that is, a smaller amplitude of the P1-N1-P2 complex, especially in the elderly group.

Table 1 shows the latency values of the analyzed cortical components (P1, N1, P2) in response to the speech stimulus /ba/ for the three types of masking, as well as the comparison between the means obtained, making it possible to observe the results in each group of participants. The latency results of the cortical components showed a decrease in this response in the masking condition with modulated noise, expressing lower means when compared to strong steady noise in the “young-adult” and “adult” groups, with statistical significance (ANOVA, post-hoc Bonferroni; *p* < 0.05) for P1’s response. In the “elderly” group, this decrease in latency results due to noise modulation was observed only in the P1 component, without statistical significance. No significant difference was found in latencies between modulated and weak steady masking, except for the elderly group.

**Table 1:** Results of the latencies of the P1, N1 and P2 components between the different noise conditions in each participant group.

Latency (ms)	Weak steady noise mean ± SD (95% CI)	Strong steady noise, mean ± SD (95% CI)	Modulated noise mean ± SD (95% CI)	ANOVA post-hoc (Bonferroni)
**Component**		**Young-adult group**		
P1	56.3 ± 11.5 (50.8–61.7)	68.9 ± 20.1 (59.4–78.3)	51.9 ± 8.0 (48.1–55.6)	***p* = 0.016**^a^,*
				*p* = 0.896^b^
				***p* = 0.003**^c^,*
N1	109.6 ± 10.0 (104.9–114.3)	118.7 ± 23.2 (107.8–129.6)	116.6 ± 12.3 (110.8–122.4)	*p* = 0.328^a^
				*p* = 0.059^b^
				*p* = 1.000^c^
P2	165.9 ± 16.1 (158.3–173.4)	174.9 ± 24.2 (163.1–186.2)	168.3 ± 12.9 (162.2–174.4)	*p* = 0.521^a^
				*p* = 1.000^b^
				*p* = 0.488^c^

**Component**		**Adult group**		
P1	56.4 ± 9.3 (52.1–60.8)	65.6 ± 10.3 (60.7–70.5)	54.8 ± 7.5 (51.3–58.4)	***p* = 0.024**^a^,*
				*p* = 1.000^b^
				***p* = 0.002**^c^,*
N1	104.6 ± 7.7 (100.9–108.2)	119.9 ± 21.5 (109.8–129.9)	116.7 ± 15.1 (109.6–123.8)	*p* = 0.022^a^
				*p* = 0.008^b^
				*p* = 1.000^c^
P2	163.8 ± 13.7 (157.4–170.3)	178.7 ± 27.4 (165.9–191.6)	173.2 ± 22.0 (162.8–183.4)	*p* = 0.108^a^
				*p* = 0.204^b^
				*p* = 0.793^c^

**Component**		**Elderly group**		
P1	59.7 ± 9.3 (55.3–64.1)	84.3 ± 18.4 (75.7–92.9)	75.8 ± 14.5 (69.0–82.6)	***p* = 0.000**^a^,*
				***p* = 0.002**^b^,*
				*p* = 0.064^c^
N1	108.6 ± 8.2 (104.7–112.4)	140.6 ± 29.6 (126.7–154.5)	145.6 ± 13.3 (139.3–151.8)	***p* = 0.000**^a^,*
				***p* = 0.000**^b^,*
				*p* = 1.000^c^
P2	186.7 ± 20.2 (177.2–196.1)	195.8 ± 34.5 (179.6–212.0)	209.8 ± 16.3 (202.1–217.4)	*p* = 0.909^a^
				***p* = 0.001**^b^,*
				*p* = 0.206^c^

ms, milliseconds; SD, standard deviation; 95% CI, 95% confidence interval.

a Comparison of means between weak and strong steady noise.

b Comparison of means between weak steady noise and modulated noise.

c Comparison of means between strong steady noise and modulated noise.

* Statistically significant difference.

Table 2 describes the amplitude values of the analyzed cortical components (P1, N1, P2) in response to the speech stimulus /ba/ for the three types of masking, making it possible to observe the results of the comparison between the means obtained in each age group. The results of the amplitudes of the three cortical components showed an increase in this response in the masking condition with modulated noise, expressing higher averages when compared to strong steady noise in the “young-adult” group, with statistical significance for the P2 component (ANOVA, post- hoc Bonferroni; *p* < 0.05).

**Table 2:** Results of the amplitudes of the P1 components. N1 and P2 between different noise conditions in each participant group.

Amplitude (µV)	Weak steady noise mean ± SD (95% CI)	Strong steady noise mean ± SD (95% CI)	Modulated noise mean ± SD (95% CI)	ANOVA post-hoc (Bonferroni)
**Component**		**Young-adult group**		
P1	5.7 ± 1.9 (4.8–6.6)	5.2 ± 1.9 (4.3–6.1)	5.8 ± 1.6 (5.0–6.6)	*p* = 0.565^a^
				*p* = 1.000^b^
				*p* = 0.373^c^
N1	5.1 ± 3.1 (3.6–6.6)	4.2 ± 3.2 (2.6–5.7)	4.4 ± 2.4 (3.2–5.5)	*p* = 0.123^a^
				*p* = 0.370^b^
				*p* = 1.000^c^
P2	5.0 ± 4.1 (3.0–6.9)	3.3 ± 2.5 (2.1–4.5)	5.4 ± 2.4 (4.2–6.5)	*p* = 0.027^a^, *
				*p* = 1.000^b^
				*p* = 0.001^c^, *

**Component**		**Adult group**		
P1	5.3 ± 1.8 (4.4–6.2)	5.4 ± 2.0 (4.4–6.3)	5.4 ± 1.7 (4.6–6.3)	*p* = 1.000^b^
				*p* = 1.000^b^
				*p* = 1.000^b^
N1	5.4 ± 2.3 (4.3–6.5)	4.1 ± 3.1 (2.6–5.6)	4.8 ± 2.4 (3.6–5.9)	*p* = 0.055^a^
				*p* = 0.368^b^
				*p* = 0.690^c^
P2	4.9 ± 2.6 (3.6–6.1)	3.4 ± 2.0 (2.5–4.4)	5.2 ± 1.8 (4.4–6.1)	*p* = 0.084^a^
				*p* = 1.000^b^
				*p* = 0.006^c^, *

**Component**		**Elderly group**		
P1	7.8 ± 3.4 (6.2–9.4)	3.8 ± 1.0 (3.3–4.3)	5.6 ± 1.5 (4.9–6.4)	*p* = 0.001^a^, *
				*p* = 0.013^b^, *
				*p* = 0.002^c^, *
N1	9.6 ± 3.8 (7.8–11.3)	3.2 ± 1.6 (2.4–4.0)	5.7 ± 2.3 (4.6–6.8)	*p* = 0.000^a^, *
				*p* = 0.000^b^, *
				*p* = 0.002^c^, *
P2	6.6 ± 2.3 (5.5–7.7)	2.3 ± 1.5 (1.6–3.0)	4.0 ± 1.5 (3.2–4.7)	*p* = 0.000^a^, *
				*p* = 0.000^b^, *
				*p* = 0.006^c^, *

µV, microvolts; SD, standard deviation; 95% CI, 95% confidence interval.

a Comparison of means between weak and strong steady noise.

b Comparison of means between weak steady noise and modulated noise.

c Comparison of means between strong steady noise and modulated noise.

* Statistically significant difference.

In the “adult” group, this increase in amplitudes was also observed in the N1 and P2 components when masking with modulated noise when compared to strong steady noise, with statistical significance for the P2 component (ANOVA, post-hoc Bonferroni; *p* < 0.05), similar to the young group.

For the “elderly” group, a statistically significant increase (ANOVA, Bonferroni post-hoc; *p* < 0.05) was observed in the amplitude of all cortical components in the modulated noise masking condition, expressing higher means when compared to the “elderly” group strong steady noise.

As with latency responses, there were no significant differences in amplitudes, that is, in the magnitude of the neural response between modulated and weak steady masking, except for the elderly group.

Table 3 shows the results of the research on electrophysiological and behavioral auditory thresholds for each group of participants, making it possible to demonstrate, in all age groups, that the masking modulation condition resulted in statistically lower thresholds (paired *t*-test; *p* < 0.05) when compared to steady masking in both hearing domains. The “elderly” group showed significantly higher electrophysiological and behavioral thresholds (ANOVA, Bonferroni post-hoc; *p* < 0.05) in both masking conditions (steady and modulated) when compared to the other age groups.

**Table 3:** Results of thresholds (electrophysiological and behavioral) and BMM in each participant group.

	Steady noise mean ± SD (95% CI)	Modulated noise mean ± SD (95% CI)	BMM mean ± SD (95% CI)	Test t paired *p-*value
**Electrophysiological threshold (dB SPL)**				
Young-adult group	49.7 ± 6.3 (46.7–52.7)	41.2 ± 5.3 (38.7–43.7)	9.5 ± 4.6 (7.0–11.4)	*p* = 0.000*,*
Adult group	52.5 ± 4.1 (50.5–54.4)	45.5 ± 3.5 (43.8–47.1)	6.7 ± 2.4 (5.6–7.9)	*p* = 0.000*,*
Elderly group	57.5 ± 5.9 (54.7–60.2)	52.7 ± 6.5 (49.6–55.8)	4.7 ± 2.5 (3.5–5.9)	*p* = 0.000*,*
ANOVA post-hoc (Bonferroni)	*p* = 0.379^a^	***p* = 0.033**^a^,*	*p* = 0.140^a^	
	***p* = 0.007**^b^,*	***p* = 0.000**^b^,*	***p* = 0.001**^b^,*	
	***p* = 0.010**^c^,*	***p* = 0.000**^c^,*	*p* = 0.050^c^	

**Behavioral threshold (dB SPL)**				
Young-adult group	59.1 ± 1.0 (58.6–59.6)	50.3 ± 1.8 (49.4–51.2)	8.7 ± 1.4 (8.1–9.4)	*p* = 0.000*,*
Adult group	59.2 ± 1.0 (58.7–59.6)	51.0 ± 1.6 (50.2–51.8)	8.1 ± 1.3 (7.5–8.7)	*p* = 0.000*,*
Elderly group	61.0 ± 1.3 (60.4–61.7)	54.7 ± 1.9 (53.8–55.6)	6.1 ± 1.5 (5.4–6.0)	***p* = 0.000**^d^,*
ANOVA post-hoc (Bonferroni)	*p* = 1.000^a^	*p* = 0.709^a^	*p* = 0.306^a^	
	***p* = 0.000**^b^,*	***p* = 0.000**^b^,*	***p* = 0.000**^b^,*	
	***p* = 0.000**^c^,*	***p* = 0.000**^c^,*	***p* = 0.005**^c^,*	

SD, standard deviation; 95% CI, 95% confidence interval; dB SPL, decibel Sound Pressure Level; BMM, Benefit of Modulated Masking.

a Comparison of means between the Young Adult and Adult groups.

b Comparison of means between the Young Adult and Elderly groups.

c Comparison of means between the Adult and Elderly groups.

d Comparison of means between steady and modulated noise.

* Statistically significant difference.

This reduction in auditory thresholds benefited by noise modulation is expressed by the BMM measurement, being observed with a statistically lower magnitude (ANOVA, post-hoc Bonferroni; *p* < 0.05) in the “elderly” group when compared to the “Young-adult” group in the electrophysiological and behavioral domains. There were no significant differences in this benefit (BMM) between the “young-adult” and “adult” groups for both hearing domains.

## Discussion

Based on the analysis of the latency results of the analyzed cortical potentials (P1-N1-P2), it was possible to verify that the decrease in these responses in the modulated masking condition indicated less interference from noise modulation in the neural synchronism for the generation of auditory potentials, more specifically the P1 potential, in the “young-adult” and “adult” groups. In the “elderly” group, the reduction in latencies due to the benefit of noise modulation was observed only in the P1 component.

Despite the masking disturbance present in the generation of cortical potentials with speech stimuli, these results demonstrate that noise modulation causes less interference in the neural auditory processing time, more specifically in the speed of encoding the electrical stimulus (represented by P1).^23^ These findings corroborate previous results regarding the effect of masking on cortical potentials, which point to a systematic reduction in response latencies in the presence of noise modulations, when compared to steady noise, referring to the existence of BMM in the analysis of this time measurement.^5^, ^14^

With regard to the results found in the study of the amplitudes of cortical potentials (P1-N1-P2), it was possible to demonstrate, in all age groups, an increase in these measurements due to masking with modulated noise, indicating less interference of this noise in the magnitude of the neural response to the speech stimulus, when compared to steady noise. This finding can be explained by the fact that modulation in noise intensity generates a lower signal/noise ratio that is favorable to the neural processing of speech.^12^

The P2 component, in turn, expressed a significant and common increase in all age groups in the face of noise modulation, which may indicate a better neuroelectric response in the speech stimulus discrimination process in the face of this masking condition, since this potential also it is related to the processing of competitive stimuli, with generators in several regions of the primary auditory and reticular cortex.^24^, ^25^

The morphology of the grand average waves, shown in Fig. 1, shows a greater disturbance in the cortical responses of the P1-N1-P2 complex in the face of strong steady noise, demonstrating a lower amplitude/magnitude of these responses, especially in the elderly group, which may indicate greater difficulty for these individuals in the neural processing of speech when faced with this type of noise.

Studies report that the amplitudes of cortical waves represent the synaptic density in the primary auditory cortex in response to sound stimuli, being influenced by the processing of competitive stimuli.^26^, ^27^ In this way, we can infer from this research that the use of steady noise determined a decrease in amplitude measurements due to the greater influence of this masking on synaptic responses to the speech stimulus, compared to modulated noise.

Regarding the research on auditory thresholds, the results of this study expressed a significant decrease in these responses in the noise modulation condition in both domains (electrophysiological and behavioral) in the three groups of participants. These findings corroborate the literature, since similar responses to reducing minimum thresholds for detection and speech perception in the face of modulated noise were obtained in comparison to steady noise in normal-hearing individuals, pointing to the presence of BMM in the generation of typically lower thresholds.^4^, ^5^

In the elderly group, obtaining higher behavioral and electrophysiological thresholds, consequently expressing a lower magnitude of BMM, compared to the “young-adult” and “adult” groups, can be justified by the relationship between the temporal processes of hearing and aging human hearing.^3^

Studies regarding the temporal processing abilities of hearing in the elderly population indicate a worsening of auditory performance, especially in situations of competitive noise, even in the presence of normal peripheral hearing, mentioning that the speed of this processing gradually decreases with the advancement of human aging.^12^, ^28^

The results of obtaining thresholds in the present study suggest that elderly individuals present inferior temporal auditory performance when compared to younger individuals and adults, and that the reduction in BMM can be attributed to a deficit in temporal resolution capacity, since this ability is related to the identification of short periods of time in front of two acoustic signals.^5^, ^29^

The findings of the present study made it possible to investigate BMM in electrophysiological and behavioral measures of hearing in different age groups, enabling the understanding of its relationship with temporal auditory processing as a function of age. It is important to highlight the need to carry out more research on this topic in different age groups with and without hearing changes, especially in elderly individuals, in order to expand the literature on the investigation of BMM under the influence of aging.

## Conclusion

The results showed that modulated masking resulted in less interference in the magnitude and processing time of the neural response to the speech stimulus, represented by increased amplitudes and reduced latency responses of cortical potentials, respectively. The elderly presented a higher threshold in both hearing domains, compared to the other participants, as well as a lower BMM magnitude, indicating a lower temporal auditory performance in this population, which may be associated with the aging process.

## Funding

This work was supported by the 10.13039/501100002322Coordenação de Aperfeiçoamento de Pessoal de Nível Superior – Brazil (CAPES) – funding code 001.

## Conflicts of interest

The authors declare no conflicts of interest.
